# Malignant proliferating trichilemmal tumor of the scalp: report of 4 cases and a short review of the literature

**DOI:** 10.1080/23320885.2022.2077208

**Published:** 2022-05-23

**Authors:** Cemal Alper Kemaloğlu, Melikgazi Öztürk, Beyza Aydın, Özlem Canöz, Orhun Eğilmez

**Affiliations:** a Department of Plastic, Reconstructive and Aesthetic Surgery, Faculty of Medicine, Erciyes University, Kayseri, Turkey; b Department of Pathology, Faculty of Medicine, Erciyes University, Kayseri, Turkey

**Keywords:** Malignant proliferating trichilemmal tumor, proliferated pilar cyst, skin tumor

## Abstract

Malignant proliferating pilar tumors are very rare adnexial lesions that can be confused with other skin neoplasms. The authors present four patients with malignant proliferating trichilemmal tumors located on the scalp. A review of the literature search for malignant proliferating pilar tumors and treatments was performed.

## Introduction

Malignant proliferating trichilemmal tumor (MPTT) is an extremely rare adnexial tumor in the malignant spectrum of proliferating pilar tumors. Histologically, it is characterized by excessive proliferation of the outer sheath epithelium of the hair follicles [1]. Usually older women are affected [2]. The lesion is often localized on the scalp, but it can also be observed more rarely on the neck, trunk, groin, lower and upper extremities [3].

Because the tumor is rare, its biological behavior is unpredictable, and it is frequently confused with SCC histopathologically, standard guidelines for treatment could not be established [4–6]. Most of the case reports in the literature have focused on the pathological features of the tumor rather than its clinical behavior and management.

In this article, 4 malignant proliferating trichilemmal tumor cases admitted to our clinic are presented. This report aims to raise awareness about this rare tumor and to contribute to the literature on its diagnosis and treatment.

## Cases

**Figure 1.**
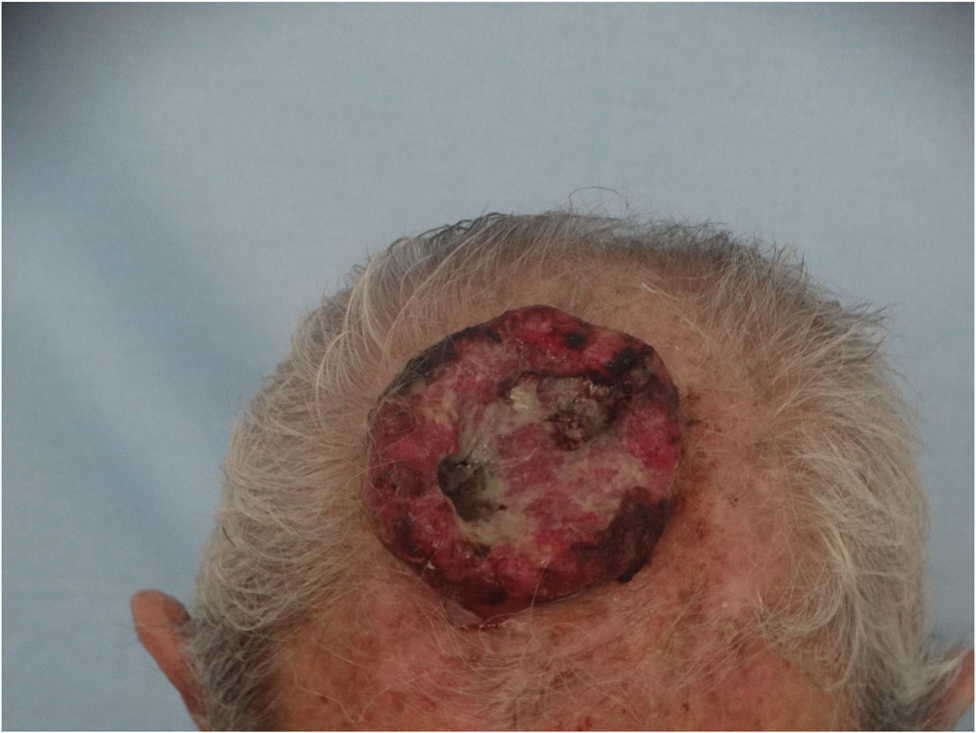
An ulcerated tumor located in the anterior of the vertex, fixed to the floor.

*Case 1*: An 85-year-old male patient applied to our clinic because of a painless lesion that had grown on the scalp for 2 years. In the examination of the patient, who had a history of coronary artery disease and gastric cancer, a 6.8 × 6.5 × 1 cm middle ulcerated, hyperemic, nonpigmented, fixed lesion was found in the anterior of the vertex (Figure 1). There was no palpable mass on neck examination. Bx was taken from the lesion and neck ultrasonography (USG) and cranial computer tomography (CT) were requested for possible neck metastasis and cranium invasion.

**Figure 2.**
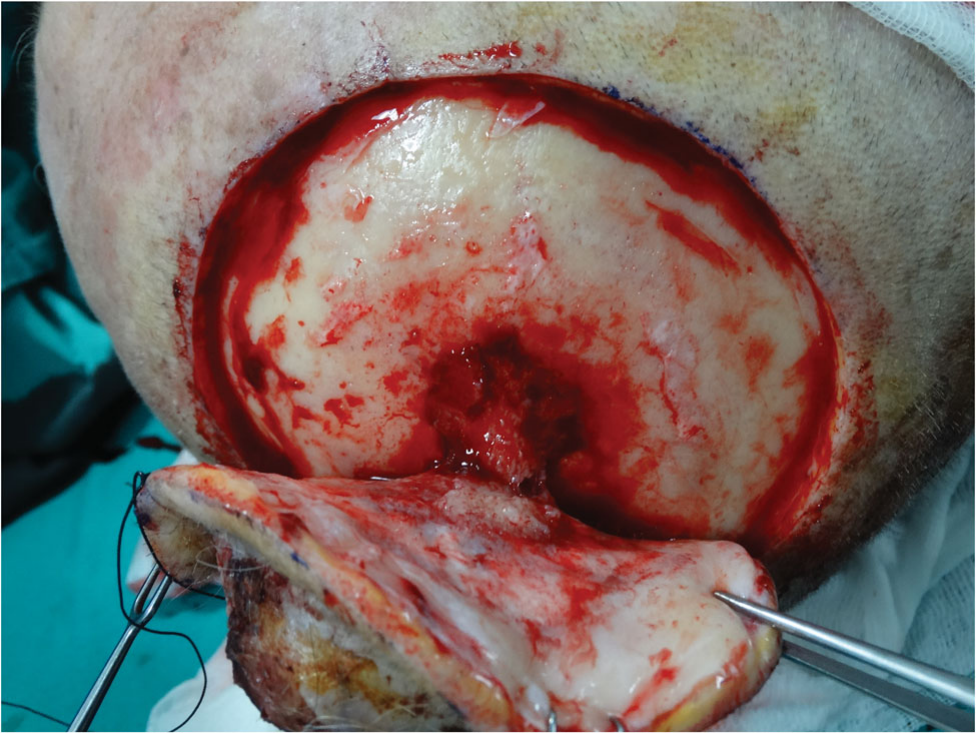
Gross invasion extending to the cranium in the tumor center.

Biopsy from the lesion was reported as poorly differentiated squamous cell carcinoma (SCC). Reactive lymph nodes smaller than 1 cm were observed in neck USG. Cranial CT revealed that the outer tabula was destroyed and the tumor extended to the diploic distance, but the inner tabula was preserved. The patient was operated under general anesthesia and the tumor was excised with a 1 cm surgical margin, along with the periosteum. It was observed that the tumor invaded the parietal bone in the center (Figure 2). Within the neurosurgery operation, the invasive bone of the patient was excised with a 1 cm surgical margin in full thickness. It was observed that the tumor continued on the dura. The tumor was curetted over the dura without causing a dural defect. The skin defect on the scalp was repaired with a right superficial temporal artery-based transposition flap. The patient was discharged without any problem on the 6th postoperative day.

**Figure 3.**
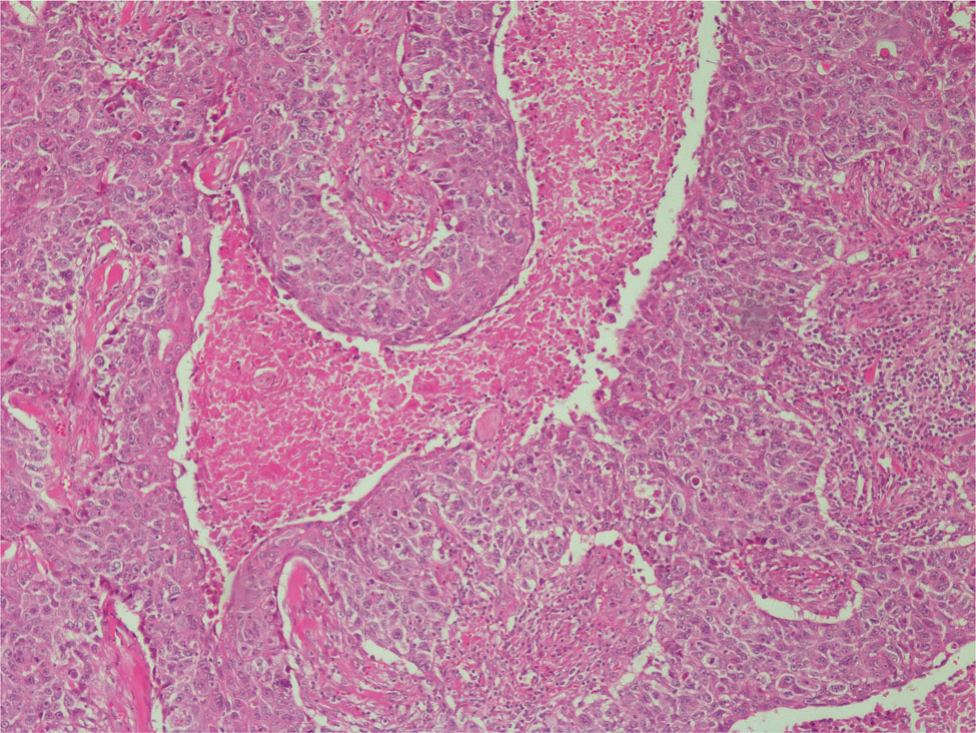
Lobular proliferation is seen. H&E; original magnification, ×100.

Histopathological diagnosis was MPTT. High grade of cellular anaplasia was noted in microscopy. Most of the tumor consisted of small cells with eosinophilic staining and high nucleus/cytoplasm ratio (Figure 3). Ki-67 was detected as 10%. Ulceration was positive in the epidermis overlying the tumor. Duramater was infiltrated with tumor cells.

The patient was employed 60 Gy adjuvant radiotherapy in 30 fractions during the postoperative period Medical oncology did not recommend chemotherapy due to age and comorbidities. The patient was followed-up every 3 months and is currently being followed up without incident at the 7th month postoperatively.

**Figure 4.**
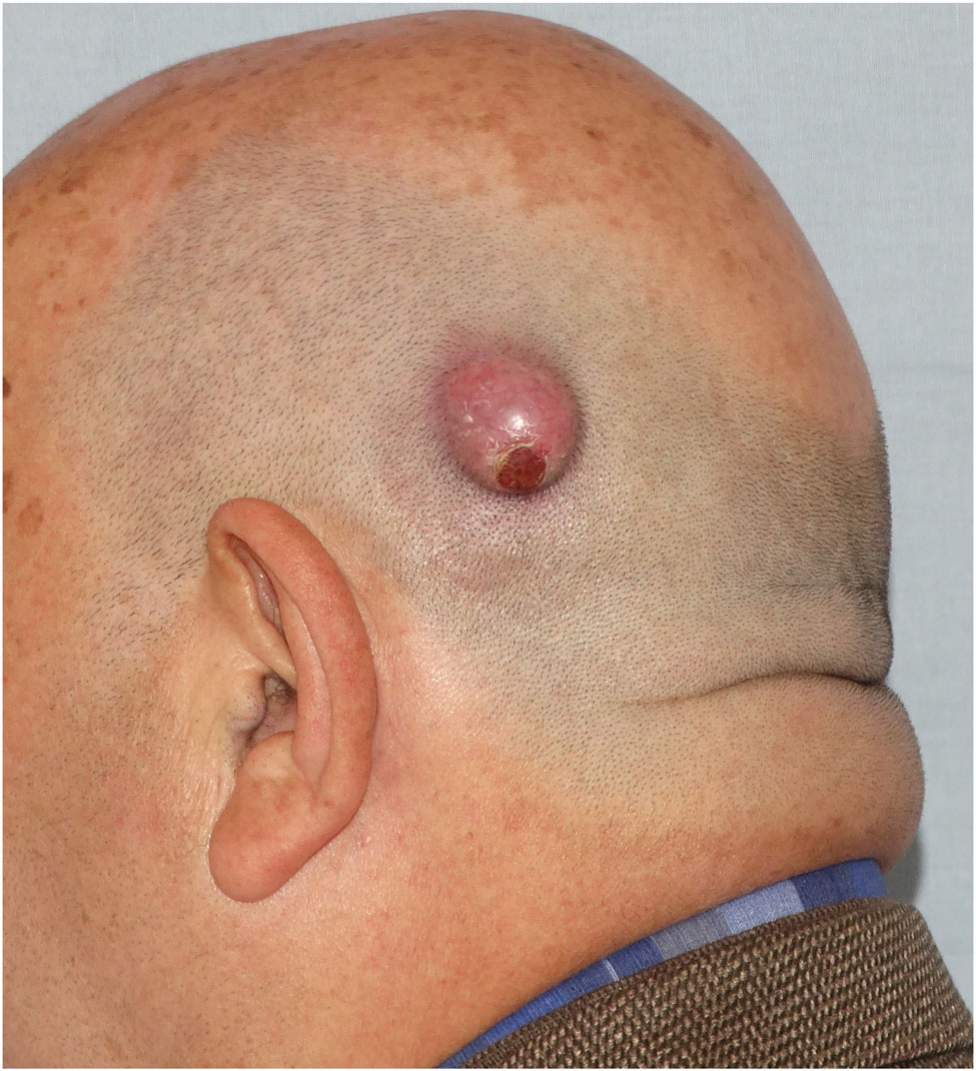
Mobile, hyperemic tumor located in the left temporal region.

*Case 2***:** A 69-year-old male patient applied to our clinic with a subcutaneous mass growing behind the left ear for 1 year. The patient had a history of hypertension, atrial fibrillation, and benign prostatic hypertrophy. In the examination, a smooth bordered, hyperemic, hair-free, and mobile 2 × 2 × 1 cm nodular lesion was detected in the left parietal region (Figure 4).

The patient was operated under local anesthesia with a preliminary diagnosis of pilar cyst. Since the mass was very close to the skin, it was excised full-thickly over the periosteum with a 2 mm surgical margin. The defect area was primarily repaired. Histopathological diagnosis was MPTT. It was reported that the tumor persisted in a lateral border. Thereupon, neck USG was taken to the patient. Pathological LAP was not detected, and the patient was re-operated and re-excision was made from the old scar line with a 5 mm surgical margin. The defect was repaired with a local rotation flap. The second pathology report of the patient came as chronic inflammatory event and foreign body granulation tissue. The patient, who was followed up by us, did not come for follow-up due to the Covid-19 pandemic.

**Figure 5.**
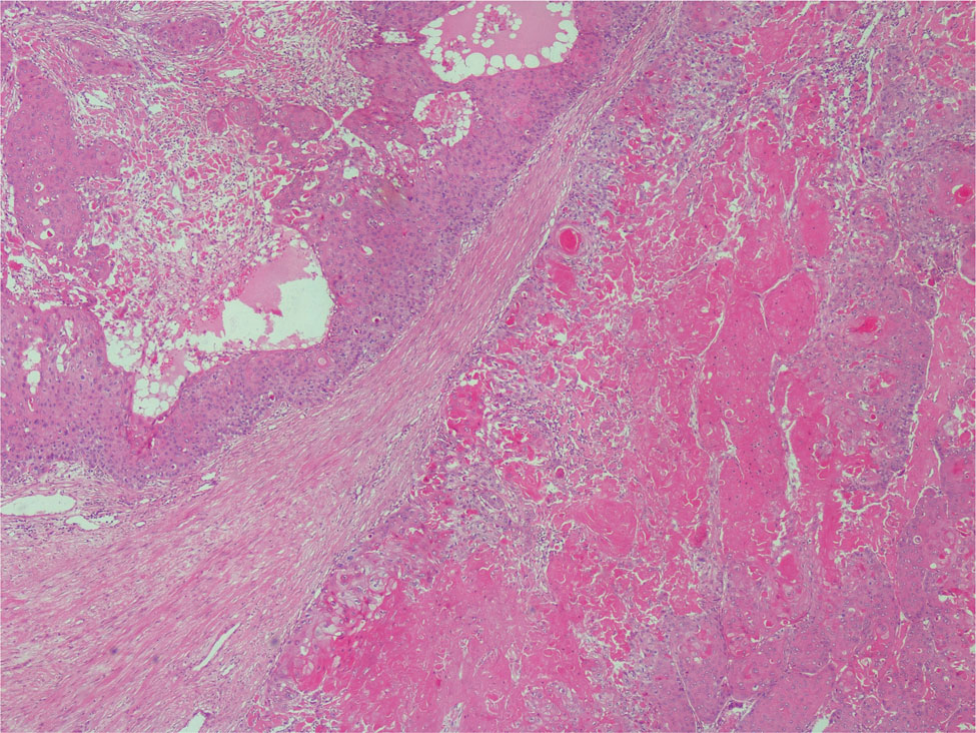
Basaloid cells with palisading in the peripheral areas of lobules are present. H&E; original magnification, ×100.

The infiltrative pattern of the tumor was noted in the first pathological examination of the patient. There was an ulcer on the overlying epidermis. The tumor cells were malignant, with eosinophilic staining, pleomorphism, dark nuclei, and large cell groups in places (Figure 5). Ki-67 was detected as 10%. The tumor tissue was nodulocystic and exhibited a trabecular growth pattern anastomosing with each other.

The patient applied to our clinic with the complaint of swelling/recurrent mass at the operation site 20 months after the second operation. Fine needle biopsy taken from the mass was reported as carcinoma metastasis. Positron emission tomography (PET) revealed multiple metastatic lymph nodes in neck zone 5 and multiple lymph node metastases in the bilateral lung. Because of stage 4, the patient was considered inoperative. The patient was administered six cycles of cisplatin, 5-fluorouracil, cetuximab regimen by medical oncology. Subsequently, radiotherapy was planned for the patient, but the patient died in the chest diseases intensive care service in the postoperative 26th months due to lung metastases.

**Figure 6.**
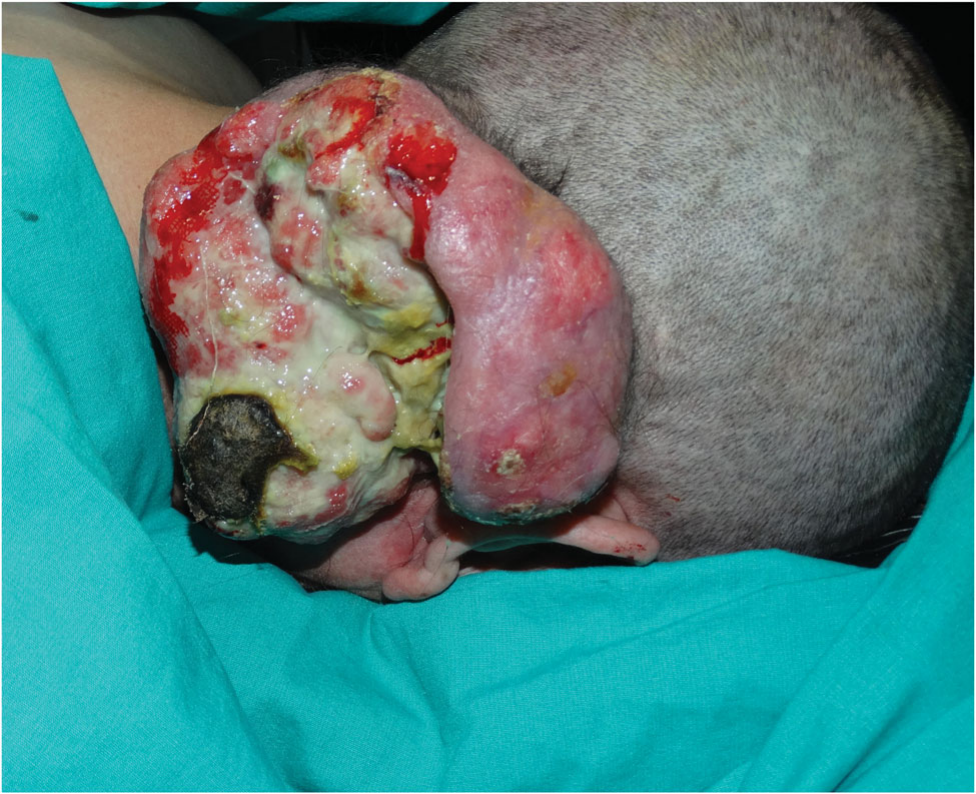
Infected and ulcerated tumor located in the right occipital region.

*Case 3***:** A 60-year-old female patient applied to our clinic with the complaint of a painful mass in the right neck region for 1 year and growing over time. Her history was unremarkable except for hypertension. In the examination, a mobile mass of 15 × 15 × 7 cm with irregular borders and purulent discharge was detected extending from the right occipital region to the neck. There was no palpable LAP in the neck (Figure 6).

Incisional biopsy was taken from the mass, and neck USG and cranial CT were also requested. From the purulent discharge, culture was taken and empiric amoxicillin + clavulanate treatment was started. Incisional biopsy result was reported as MPTT. Reactive lymph nodes were detected in neck USG. It is seen in cranial CT that the tumor caused edema in the surrounding soft tissues without causing bone erosion. The patient was operated under general anesthesia. The tumor was excised with a surgical margin of 1 cm over the periosteum, sternocleidomastoideus, and trapezius muscles. The defect area was repaired with a partial thickness skin graft taken from the posterior thigh. When the graft dressing was opened on the 5th postoperative day, it was found that the graft was vital, but there was purulent discharge in one focus. Intravenous piperacillin + tazobactam treatment was started, including Pseudomonas aeruginosa grown in the first culture. On the 10th day of antibiotic treatment, the infection was resolved and the patient was discharged.

**Figure 7.**
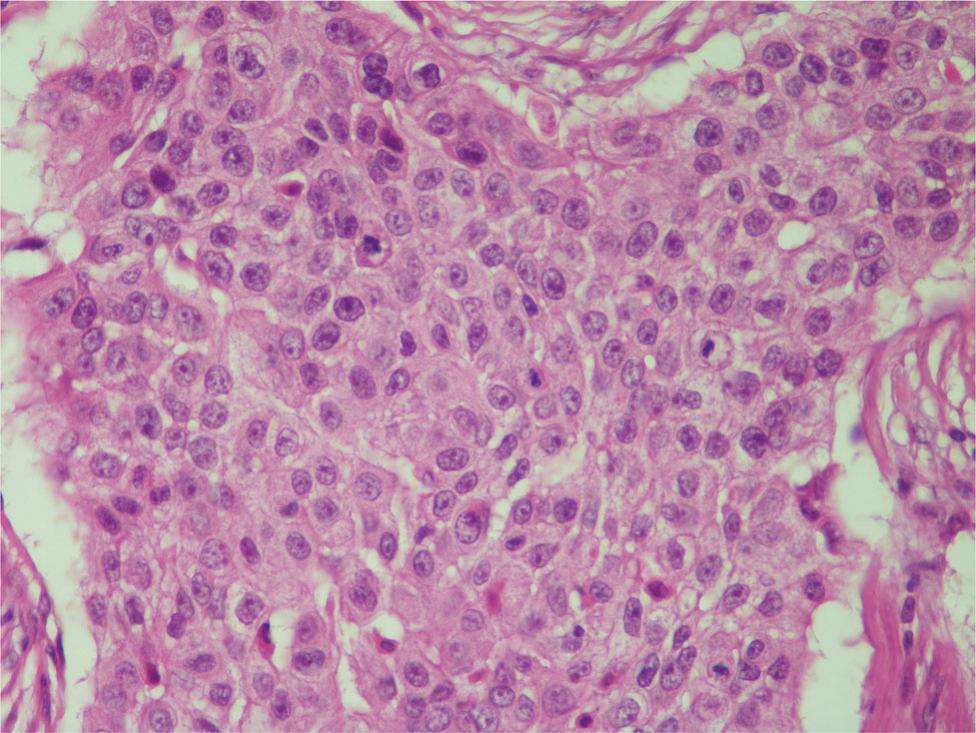
Marked anaplasia and atypical mitosis are seen. H&E; original magnification, ×400.

In the histopathological examination of the tumor, lobular, and trabecular structures including cystic and amorphous eosinophilic keratin were noted. Tumor cells showed irregular nuclear contours, hyperchromasia, and high mitotic activity with atypical mitotic figures (Figure 7). Ki-67 was found to be 4%.

The patient was employed 50 Gy adjuvant radiotherapy in 25 fractions during the postoperative period. The patient was followed-up every 3 months and is currently being followed up without incident at the 38th month postoperatively.

**Figure 8.**
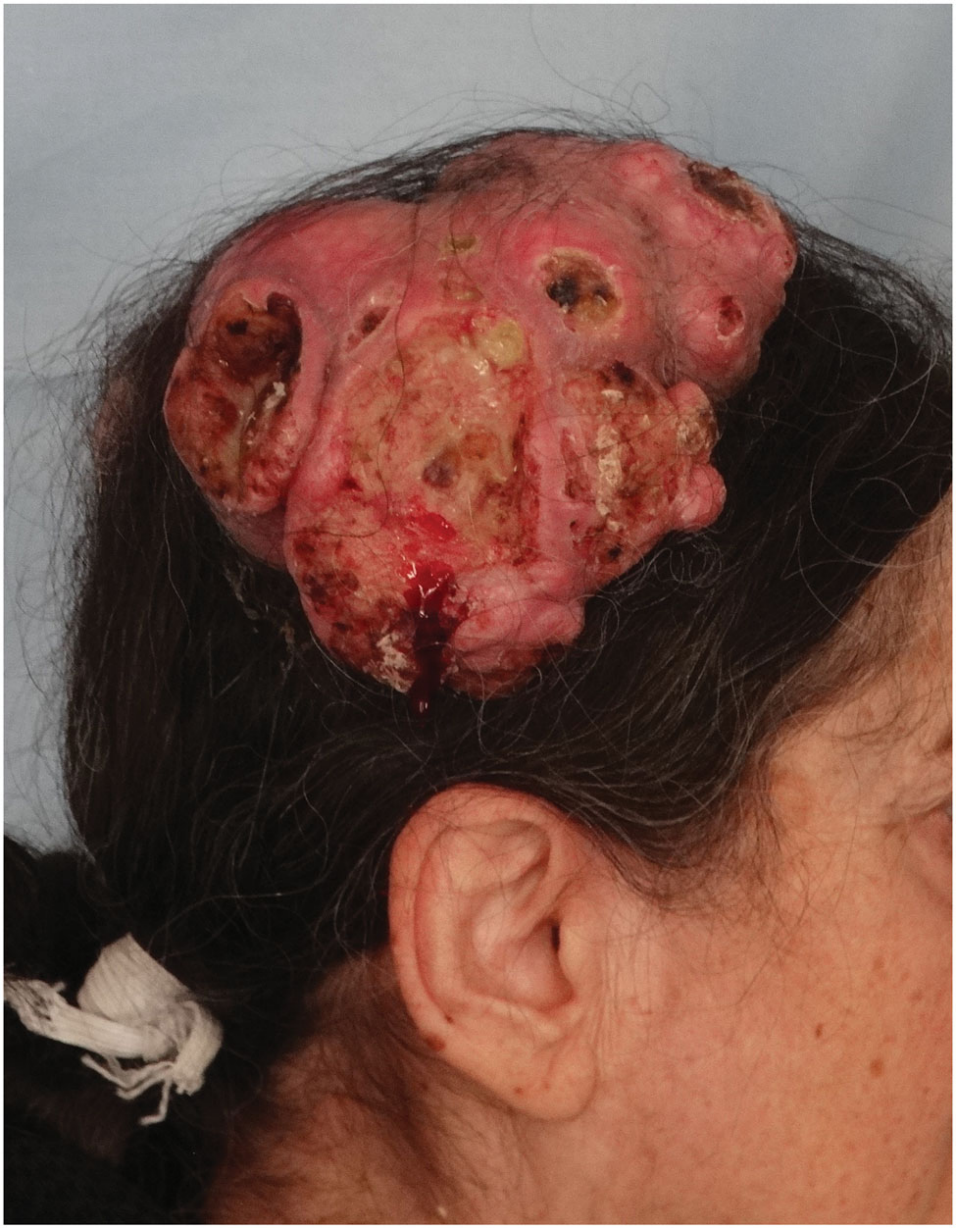
Tumor located in the right parietal region, with multiple ulcerated areas on it.

*Case 4***:** A 55-year-old female patient applied to our clinic with a painless mass on the scalp that had been growing for 1.5 years. The patient had a history of diabetes, hypertension, breast, and stomach cancer. The patient reported that she had surgery for cancer treatment 5 years ago and received adjuvant chemotherapy. In the examination, a mobile, multilobulating, hair-free mass of 6.5 × 5 × 3 cm with irregular borders and ulcerations in places, was detected in the right temporoparietal region (Figure 8). There was no palpable LAP in the neck.

An incisional bx was taken from the mass. Because of past cancer history, neck USG, cranial CT, and also PET were requested. The biopsy result was reported as MPTT. Neck USG was unremarkable. Bone invasion was not observed in cranial CT. In PET-CT, no recurrence/residue/metastasis was detected. Surgery was recommended to the patient, but the patient refused it due to comorbidities.

The patient was admitted to our clinic again due to the significant enlargement of the lesion after 9 months. The new size of the lesion is measured as 11 × 9 × 3.5 cm. The patient’s radiological examinations were repeated and a suspicious lymph node was detected in the right postauricular region. Fine needle aspiration biopsy taken from this lymph node was clean. The patient’s tumor was excised over the periosteum with a 1 cm surgical margin under general anesthesia. The defect area was repaired with a partial thickness skin graft taken from the posterior thigh region. The patient was discharged without any problem in the postoperative follow-ups.

**Figure 9.**
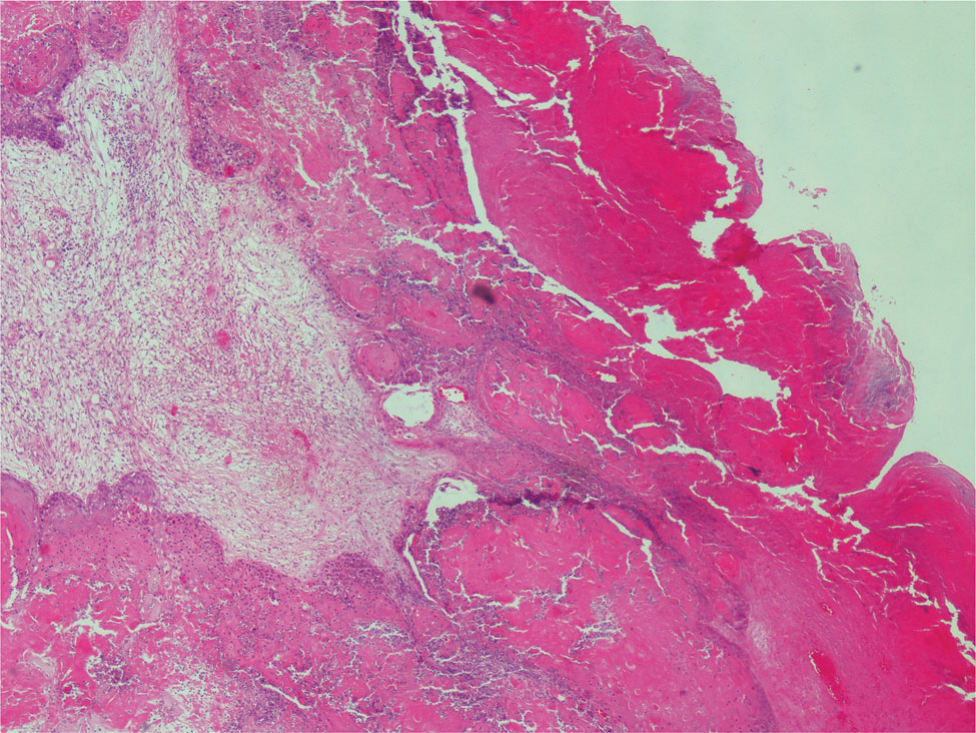
On low power, pushing border and trichilemmal keratinization. H&E; original magnification, ×40.

Histopathological examination revealed proliferative keratinocytes surrounding the trichylememal type keratinous material (Figure 9). High mitotic activity and nuclear polymorphism were observed in tumoral cells. Ki-67 was positive at 20%.

The patient was employed 50 Gy adjuvant radiotherapy in 25 fractions during the postoperative period. The patient was followed-up every 3 months and is currently being followed up without incident at the 10th month postoperatively.

## Discussion

Proliferating trichilemmal tumor (PTT) is the proliferating form of pillar cysts. This dermal neoplasm was first described by Wilson-Jones in 1966 and stated that it has a histological capacity that mimics SCC [7]. PTTs are formed by increased epithelial proliferation in pilar cysts and are usually seen where hair follicle growth is high. The tumor usually appears on the scalp in the fourth to eighth decade of life and mostly affects women. The progression of the tumor is slow but locally invasive. Ulcers may appear over time [8]. Although PTTs are considered benign, there are cases in which SCC development has been reported in the literature [9]. Treatment is almost always surgical excision.

On the other hand, MPTT is a less often adnexial cancer originating from the outer sheath epithelium of hair follicles. MPTTs constitute less than 0.1% of skin cancers [10]. Although it usually develops over existing pilar cysts, they can also occur as a denovo without a precursor lesion [11]. They are generally seen on the sun-exposed areas and especially on the scalp in elderly women. Since it originates only from the terminal hair root, it is very unlikely to develop from lanugo in bald men or from nonterminal hair follicles on the body [12].

In MPTT histopathology, invasion of surrounding connective tissue, high mitosis rate, necrosis, nuclear atypia, cellular pleomorphism, dyskeratosis, absence of granular layer and lobular proliferation of glycogenized clear cell squamous epithelium are frequently observed [13]. Also, Herrero et al. reported that in the diagnosis of MPTT, cellular aneuploidy and CD34 loss are important markers [14]. The separation from the surrounding stroma by a sharp demarcation line is an important feature in SCC differentiation [15]. In addition, p53 and Ki-67 markers in immunohistochemical examination are guiding in predicting the aggressiveness of the tumor (low grade-high grade). Decreased expression of p53, which is responsible for the repair of DNA damage, and increased expression of Ki-67, which indicates a high rate of mitosis in the cell, have been associated with malignant transformation and high recurrence rates [16–18].

The diagnosis of MPTT is usually made by clinical observation and biopsy. In the differential diagnosis, especially PTT, SCC and pilomatrix carcinoma should be kept in the foreground. The most confused entity is the PTT. The similarity of PTTs that present themselves as exophytic, multilobule, and occasionally ulcerated masses with MPTT may cause delays in diagnosis. As a matter of fact, it has been noted that some cases reported as PTT in the literature later relapsed in the same region and these recurrences were later diagnosed as MPTT [19–21]. This suggests that most of the recurrent PTT cases may actually be missed MPTT. Noto [22] approached this situation from a different viewpoint and claimed that all PTTs should actually be considered as low-grade adnexial tumors. Again, especially SCC should be considered in the microscopic differential diagnosis. Trichilemmal keratinization and complex cellular architecture observed in the histopathology of MPTTs may cause them to be frequently confused with SCC. For discrimination, immunohistochemical staining is usually required [3,23].

Another manifestation that can be confused with MPTT is pilomatrix carcinoma. It is a rare malignant hair follicle tumor that typically presents as a nontender, firm dermal swelling on the head and neck regions with vary in size from 1 to 10 cm. Histologically, pilomatrix carcinomas show proliferating atypical basaloid cells with an infiltrating border [24]. Necrotic areas and frequent mitoses are often seen. Unlike MPTT, multiple lobulated and bosselated expansive masses of squamous epithelium was not observed. Pilomatrix carcinoma composed mostly basaloid cells, shows infiltrating border and marked nuclear pleomorphism with prominent nucleoli. Most studies recommend complete excision with 5–30 mm excision margins to prevent recurrence [25,26]. Adjuvant radiation therapy should be generally reserved for recurrent disease or residual macroscopic disease. Finally, trichilemmal carcinoma, which can cause confusion because of the name resemblance, should also be mentioned. This tumor is a completely separate entity from the MPTT. Trichilemmal carcinomas develop from trichilemmomas and their clinical course is not as aggressive as MPTT [27].

Radiology is not very useful in the diagnosis of MPTT. On the other part, some radiological findings observed in selected cases may help the diagnosis. These are dimensions greater than 5 cm, irregular borders, presence of invasion, and an increase in the solid/cystic component ratio [28,29].

It is difficult to make accurate predictions about the clinical course since most of the MPTT cases in the literature have been reported as small case series. It is known that the tumor exhibits local invasion, can cross the surrounding tissue planes, and even exhibits intracranial extension, causing serious morbidity and mortality. Ye et al. [2] reported the rates of local recurrence and regional lymph node metastasis for lesions located on the scalp as 3.7% and 1.2%, respectively, and as 6.6% and 2.6% for lesions located outside the scalp in their MPTT meta-analysis of 185 patients. On the other part, both Ye and Sau [30] found the metastasis rates to be 25% in their series and reported that the tumor was aggressive towards SCC. Again, metastases were seen at the time of diagnosis in some series and 10 years later in some series [15,30,31]. In this context, the true metastasis rates of MPTTs are uncertain.

A guideline with a high level of evidence could not be established for their treatment since MPTTs are seen very rarely. Lymph node examination, head and neck USG, CT, and in selected cases PET scan are recommended for pre-treatment staging. Surgical excision with a margin of 1 cm is the main step in the treatment of almost all MPTTs [2,32,33]. In addition, some researchers have suggested Mohs micrographic surgery, taking into account the microscopic spread of the tumor [34,35]. Some researchers have added sentinel lymph node sampling, radiotherapy, and chemotherapy to their treatment protocols in addition to local excision [36–39]. However, there are no randomized controlled studies on the situations in which these treatment modalities will be applied or their effectiveness. As a matter of fact, the effectiveness of the CAV (cisplatin, adriamycin, vindesine) regimen used in the treatment of SCC in the treatment of MPTT is quite low [37]. On the other hand, it has been reported that radical radiotherapy can be a treatment alternative in patients with advanced age and high surgical complications [40]. Finally, considering the recurrent nature of the tumor, the importance of close follow-up, any growth on the surgical area, and lymph node examination for long periods has been emphasized in almost all studies.

All of the lesions of the 4 locally advanced MPTT patients mentioned here are localized to the scalp. In all patients, the tumor developed denovo. Stories are long-termed. Due to the late admission of the patients to the hospital, the tumor showed local invasion in some patients and the treatment became complicated. Another important issue is that although there is significant growth in tumor size and invasion into tissue planes, none of them has lymph node metastasis at the time of diagnosis. However, the fact that a patient with a tumor size of 2 cm delays the follow-up and presents with systemic metastases in the late period, suggests that the tumor may be of an aggressive nature independent of the diameter. Adjuvant radiotherapy was recommended for three patients with local invasion or Ki-67 index higher than 10%, and recurrence could be prevented in these patients. However, the development of multiple metastases in case 2, which did not show local invasion and had a low Ki-67 index, suggests that adjuvant radiotherapy should be applied to all patients.

The limitations of our study were small patient groups, short follow-up, and lack of molecular studies. In addition, the late presentation of almost all of the patients presented here makes it difficult to understand the early prognosis of the tumor. At this point, there is a need for large case series with long-term follow-up to understand the complexity of the tumor behavior.

## Conclusion

MPTTs are rare adnexial tumors that require a multidisciplinary approach to diagnosis and treatment. It is not easy to correlate the histopathological features of the tumor with its clinical presentation. To create treatment algorithms with a sufficient level of evidence, there is a need for increased case reports and comparative long-term results of different treatment options. Until these happen, close clinical follow-up is the most effective way to detect recurrence and metastases early.
